# Examination of sequence variations in partial mitochondrial 12S gene amongst damselfish species as references for DNA barcoding

**DOI:** 10.3897/BDJ.12.e126744

**Published:** 2024-08-15

**Authors:** Takumi Yamamoto, Katsunori Tachihara, Mamoru Toda

**Affiliations:** 1 Graduate School of Engineering and Science, University of the Ryukyus, Nishihara, Okinawa, Japan Graduate School of Engineering and Science, University of the Ryukyus Nishihara, Okinawa Japan; 2 Laboratory of Fisheries Biology and Coral Reef Studies, Faculty of Science, University of the Ryukyus, Nishihara, Okinawa, Japan Laboratory of Fisheries Biology and Coral Reef Studies, Faculty of Science, University of the Ryukyus Nishihara, Okinawa Japan; 3 Tropical Biosphere Research Center, University of the Ryukyus, Nishihara, Okinawa, Japan Tropical Biosphere Research Center, University of the Ryukyus Nishihara, Okinawa Japan

**Keywords:** coral reefs, DNA database, MiFish, Pomacentridae, Ryukyu Islands

## Abstract

Accurate species identification, based on DNA barcoding, can be achieved when sufficient sequence variations are present amongst species in the sampled marker. In general, the ability to discriminate species decreases with shorter sequences; however, shorter regions have a merit in amplification success by the polymerase chain reaction. In either case, it is important to investigate sequence variations amongst species before barcoding to understand its reliability and limitations. In this study, we investigate how accurately short, but hypervariable portion of the mitochondrial 12S ribosomal RNA (12S) gene (MiFish region with approximately 180 bp) is used to identify each species in diversified pomacentrid fishes compared with the longer region of the same gene (approximately 750 bp). We prepared three datasets with 301 sequences of the MiFish region for 150 species, the same 301 of sequences of the longer 12S region and 476 sequences of the MiFish region for 183 species. Neighbour-joining (NJ) analyses and genetic distance analyses revealed several indistinguishable pairs of species in these DNA regions. Although the number of such pairs was larger in the MiFish region, 83.6% (153 of 183) of species possessed respective unique sequences even in the MiFish region (versus 96.0% [144 of 150 species] in the longer 12S region). A part of indistinguishable pairs of species might have caused by mitochondrial DNA introgressions and taxonomically unresolved problems. Our analysis clarified the effectiveness and limitations of species identification using DNA barcoding for Pomacentridae and the sequences we provided here contribute to the expansion of references for pomacentrid mitochondrial 12S sequences.

## Introduction

DNA barcoding is a rapid species identification tool that uses sequences of the short fragment of DNA (Hebert et al. 2003, Hebert and Gregory 2005). Comprehensive sequence reference is vital for accurate and adequate species identification using DNA barcoding, in which a given sequence in query would demonstrate high consistency with one of the references. When sufficient sequence references are available, DNA barcoding serves as an excellent identification tool, if interspecific variation is greater than intraspecific variation (Meyer and Paulay 2005, Stoeckle and Thaler 2014). Nevertheless, this condition may be violated in a group that has a short elapsed time from the common ancestor or that has an incomplete reproductive isolation causing genetic introgressions (Ballard and Whitlock 2004, Rubinoff et al. 2006). To apply DNA barcoding to such a group, it is necessary to examine in advance whether there are certain degrees of sequence difference in the target DNA region amongst all pairwise combinations of the species involved.

Taxonomists are not the only potential users of DNA barcoding; this technology has become useful for various fields of biology (Joly et al. 2014, Kress et al. 2015). Especially, recent popularisation of next-generation sequencers enables researchers to utilise environmental DNA (eDNA) metabarcoding analysis, by which we could understand the species composition in a given region from water, soil and aerosol samples (Ruppert et al. 2019). In conventional DNA barcoding, a partial sequence with approximately 650 bp of the mitochondrial cytochrome oxidase subunit I gene (COI) has been used and a large amount of relevant data has been compiled in the Barcode of Life Data (BOLD) Systems across animals (Ratnasingham and Hebert 2007). In contrast, much shorter DNA regions (typically < 200 bp) are used in eDNA metabarcoding, because a short fragment of DNA is much easier to amplify in the polymerase chain reaction (PCR), especially when the template DNAs are degraded (Ficetola et al. 2008, Shokralla et al. 2012, Freeland 2017). For species discrimination using short sequences in DNA barcoding, hypervariable regions with conservative flanking regions are required and the utilisation of mtDNA genes other than COI has been proposed (Collins and Cruickshank 2013, Zhang et al. 2020). A partial sequence of the mitochondrial 12S ribosomal RNA gene (12S) is such a region and often used to analyse eDNA in water samples (Miya et al. 2015, Valentini et al. 2016, Taberlet et al. 2018).

For eDNA metabarcoding for fishes, Miya et al. (2015) proposed an excellent highly versatile primer pair termed MiFish, which was designed for amplifying a hypervariable region with an average length of 172 bp within the 12S rRNA gene. The performance of MiFish primers is apparently high enough to detect species of most groups of fish and these primers have been used for biodiversity monitoring of fishes in various aquatic environments in and around six continents worldwide (Miya et al. 2020). Nonetheless, many authors (Miya et al. 2015, Morita et al. 2019, Takeuchi et al. 2019, Miya et al. 2020) mentioned that sequence variations in the region amplified using MiFish primers are insufficient for accurate species identification in several groups of fish, including tunas (*Thunnus*), eels (*Anguilla*) and salmonid species.

Damselfishes (family Pomacentridae) are a major component of the coral reef fishes (Depczynski et al. 2007). Currently, 430 species are recognised in this family (Fricke et al. 2024) and new species are still described at a growing pace (e.g. Allen and Allen (2021), Allen et al. (2022), Prokofiev and Astakhov (2023)). This family contains relatively recent radiated lineages (Cooper et al. 2009) and it is believed that there are no diferences in sequence differences between some combinations of species in these groups, especially in a short fragment of DNA. Therefore, we explored whether damselfish species could be discriminated using short mitochondrial DNA sequences. In this study, we determined the sequences of approximately 40 species of damselfish (Pomacentridae) collected in the coral reef of the Ryukyu Islands, Japan and investigated sequence variations in the MiFish region amongst pomacentrid fishes, based on large datasets compiled with other available sequences.

## Material and methods

### Collection of sequences

We used 44 pomacentrid species of 12 genera that we collected in the shallow waters of coral reefs around Okinawa-jima Island of the Ryukyu Archipelago, Japan, for sequence analysis. We examined more than one specimen for each species whenever possible, considering possible intraspecific variations (Table 1). Before genetic examinations, we attempted accurate species identification, based on morphology for each specimen following (Nakabou 2013, Allen et al. 2015, Wibowo et al. 2017, Wibowo et al. 2018 and Tang et al. 2021). The pectoral fins on the right side were collected from the specimens and stored in 99% ethanol until use. The voucher specimens were fixed with 10% formalin and transferred to 99% ethanol and representative specimens were deposited in the Ichthyological Collection of the Okinawa Churaumi Aquarium (OCF-P10591 to OCF-P10681). The remaining specimens are stored in the authors' personal collection shown as POM in Table 1.

1Total DNA was extracted from the tissue samples using DNeasy Blood & Tissue Kit (Qiagen), according to the manufacturer’s instructions. A fragment of the first half of the mitochondrial 12S ribosomal RNA (12S) gene (approximately 750 bp), which covered the entire length of the MiFish region inside, was amplified by standard PCR using Takara Ex Taq (Takara). The primers used for PCR were L709 (5ʹ-TACACATGCAAGTCTCCGCA-3ʹ) and H1552 (5ʹ-ACTTACCGTGTTACGACTTGCCTC-3ʹ) (Inoue et al. 2000). The cycling profile in PCR amplification consisted of an initial denaturation step at 94℃ for 90 s followed by 30 cycles of denaturation at 94℃ for 30 s, annealing at 49℃ for 30 s, extension at 72℃ for 45 s and final extension at 72℃ for 5 min. The PCR products, after confirmation using 1% agarose gel electrophoresis, were purified by PEG precipitation and then cycle-sequenced using BigDye v. 3.1 (Applied Biosystems) with a cycling profile of initial denaturation at 96℃ for 60 s followed by 25 cycles of denaturation at 96℃ for 10 s, annealing at 50℃ for 5 s and final extension at 60℃ for 4 min. The direct sequence was commissioned to an external company (Macrogen Japan). The sequences we determined are available at the International Nucleotide Sequence Database (INSD) (accession numbers LC814855 to LC814984). In addition to the sequences we determined, homologous sequences of pomacentrid fish were obtained from INSD. We first obtained sequences of the first half of the mitochondrial 12S gene (725–773 bp) for 154 species. These sequences were also used to generate a separate dataset of the MiFish region (166–199 bp) by cutting off redundant regions from the sequences. We further obtained sequences for 39 species for which only sequences of the MiFish regions are available in the INSD. By combining with the sequences we determined by ourselves, we obtained 330 longer sequences of the first half of the 12S region (725–773 bp) for 165 species and 523 sequences of the MiFish region for 194 species (Table 1). Furthermore, 20 sequences of a possibly undescribed species of the genus *Pomacentrus*, which was referred to as “Minamiiso-suzumedai” by Motomura (2020), were included in our data. In this study, it was treated as “*P.* sp. minamiiso”.

### Preparation of datasets

To detect sequences with unreliable labels due to various errors (Claver et al. 2022), we tentatively constructed Neighbour-joining trees for sequences of the longer 12S region and those of the MiFish region, based on the Tamura-Nei distance after sequence alignment using the MEGA-X software (Kumar et al. 2018). The pairwise distances for conspecific sequences and those for interspecific, but congeneric, sequences were calculated for the longer 12S and MiFish sequences using the MEGA-X software. Then, the sequences that were assigned to strange positions in the trees and those demonstrating apparently low sequence similarity to other conspecific or congeneric sequences were scrutinised and excluded when they were uncertain. The species that were subjected to taxonomic revisions after sequence deposition to INSD were carefully consulted and scientific names were replaced with others in 36 cases according to the latest taxonomic system described by Tang et al. (2021) (Table 1).

After removing erroneous sequences, we prepared three datasets. The first was the “longer 12S dataset (725–773 bp)” that consisted of 301 sequences for 150 species (8.8% of the equences we obtained from INSD was excluded after scrutinisation; Table 1), of which 53 species have more than one sequence. It covered 70.2% (80/114) of pomacentrid species known from the Japanese waters and 36.1% of species known in the world. The second was the dataset of the MiFish region (166–199 bp) consisting of the same 301 sequences for the 150 species that was generated by cutting off redundant regions from the first dataset. The third dataset was of the MiFish region, but including additional sequences retrieved from INSD. This dataset consisted of 476 sequences for 183 species (9.3% of the sequences we obtained from INSD was excluded; Table 1), with 87 species having more than one sequence and covered 89.5% (102/114) of Japanese species and 43.8% of species in the world.

### Evaluating the ability of DNA barcoding

NJ analyses and genetic distance calculations were performed for the three datasets to investigate the capability of the two DNA regions (the longer 12S and the MiFish regions) for species identification. We first evaluated their capabilities according to placement of the conspecific sequences in the NJ trees. The NJ trees, based on the Tamura-Nei distance, were drawn and subjected to the bootstrap analysis with 1,000 pseudoreplications using the MEGA-X software. Ratnasingham and Hebert (2013) recognised the following four categories in evaluation of the performance of DNA barcoding of a given DNA region, based on recognition of the operational taxonomic unit (OTU) using the NJ tree framework: 1) Match, in which all sequences of a given species were placed collectively and exclusively in a single OTU, indicating that the barcoding would be successful for that species; 2) Split, in which the sequences of a given species were assigned to more than one OTU, but not intermixed with other species, indicating that barcoding would be a failure; 3) Merge, in which all sequences of a given species were placed in a single OTU together with another species, indicating that barcoding would be a failure; and 4) Mixture, in which the sequences of a given species were assigned to more than one OTU and at least one sequence is shared by another species, indicating that barcoding would be a failure. We adopted these criteria in evaluations of the capabilities of the longer 12S and MiFish regions for the pomacentrid species.

It is a fact that the NJ analyses approach is not applicable to species for which only a single sequence was included in the datasets, although we could consider barcoding a failure when the single sequence of a given species is shared by another (i.e. equivalent to “Merge” in the abovementioned four categories). In other cases, we focused on the genetic distance of a given species with its most closely-related species compared with the genetic distance generally observed within a species. Uncorrected p-distance was calculated for combinations of the relevant sequences using the MEGA-X software.

For setting an upper threshold of genetic distance within a species, we referred to genetic distances within each of the 53 and 87 species for which more than one sequence is available in the longer 12S dataset and the third dataset of the MiFish region with the larger samples. Our analyses demonstrated that the maximum intraspecific genetic distances were < 0.01 for 86.8% (longer 12S) or 80.5% (MiFish) of the species, respectively (see “Results”). Thus, we tentatively included 0.01 in the genetic distance as a threshold and judged a given species as “success” for barcoding when the genetic distance between that species and its closest relatives was > 0.01.

## Results

### Evaluation of sequence difference, based on the NJ analysis

**Figure 1.**
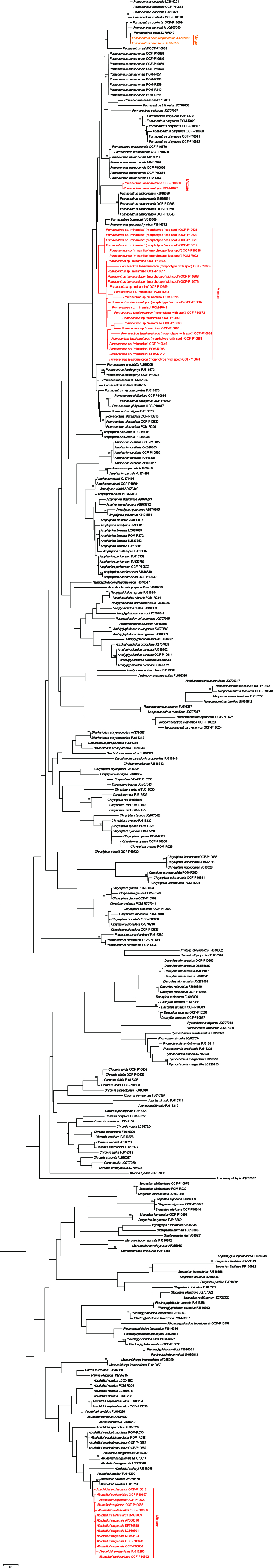
The Neighbour-joining tree constructed, based on the longer 12S dataset for 301 sequences of 150 pomacentrid species. Identical sequences are not merged and, thus, all the 301 sequences appear as terminal OTUs. Those sharing identical sequences are expressed as branch length of zero. The bootstrap values are shown for species were judged "Match".

**Figure 2.**
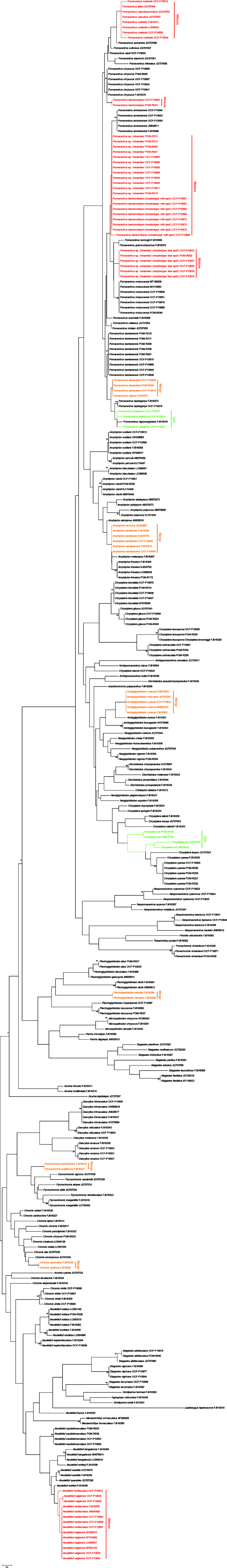
The Neighbour-joining tree constructed, based on the MiFish dataset for 301 sequences of 150 pomacentrid species. The bootstrap values are shown for species were judged "Match".

**Figure 3.**
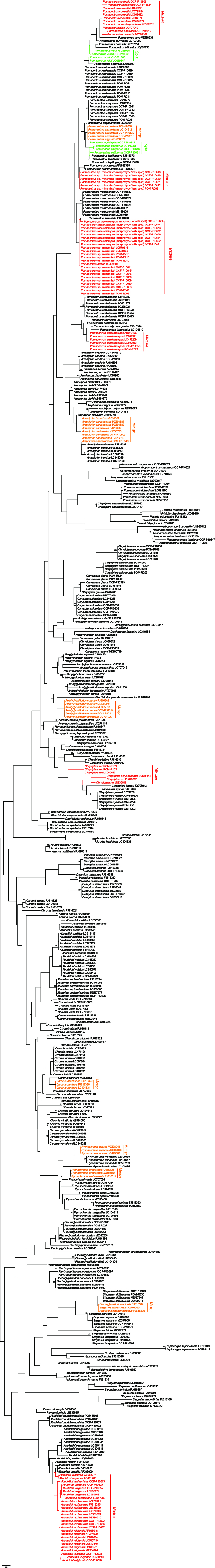
The Neighbour-joining tree constructed, based on the third dataset of the MiFish region for 476 sequences of 183 species. The bootstrap values are shown for species were judged "Match".

The NJ trees constructed, based on the longer 12S and MiFish datasets, are depicted in Figs. 1–3. In the longer 12S NJ tree, conspecific sequences of 49 of the 53 species for which more than one sequence was available exclusively formed respective clades and were judged “Match” (Fig. 1). The bootstrap values for these 49 clades were 64–99 (96.7 on average). Sequences of the remaining four species were classified as “Mixture”. In addition to these, 2 of 97 single-specimen species shared identical sequences with other species and were classified as “Merge”.

In the NJ tree constructed, based on the MiFish dataset for the same samples, the sequences of 41 species exclusively formed respective clades (Fig. 2). Of the remaining 12 species, two were classified as “Split,” four were classified as “Merge” and six were classified as “Mixture”. In addition to these, 12 single-specimen species shared identical sequences with other species and were classified as “Merge”.

Fig. 3 shows the NJ tree constructed, based on the third dataset with 476 MiFish sequences for 183 species. Conspecific sequences of 71 of the 87 species for which more than one sequence was examined exclusively formed respective clades. The bootstrap values for these clades were 37–100 (85.7 on average). Of the remaining 16 species, two were classified as “Split”, seven were classified as “Merge” and seven were classified as “Mixture” (Table 2). In addition to these, 13 of 96 single-specimen species shared identical sequences with other species and were classified as “Merge”.

### Evaluation using genetic distance

**Figure 4.**
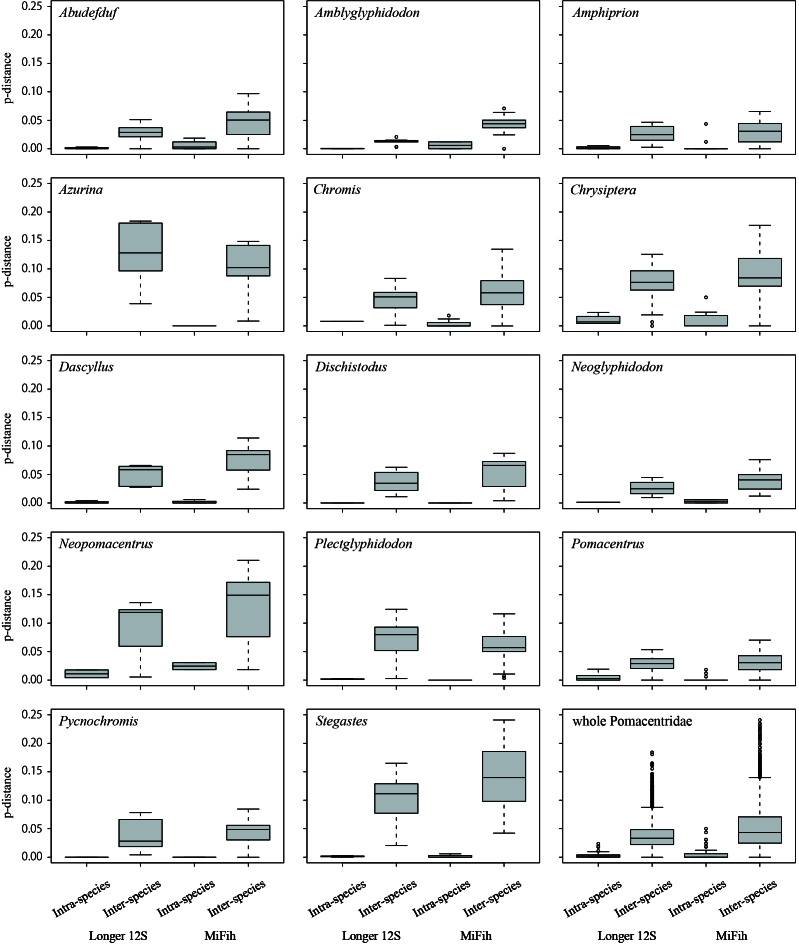
Intraspecific and interspecific variations in the sequences of the longer 12S and MiFish regions for each genus of Pomacentridae.

Fig. 4 shows an overview of the genetic distance at the intraspecific and interspecific levels. In the longer 12S dataset, the average number of base substitutions amongst conspecific sequences was 3.3 bp (equivalent to 0.004 in the uncorrected p-distance), ranging from 0 to 20 bp (0–0.026 in the genetic distances). Of the 53 species for which sequences of more than one individual were available, 47 species (86.8%) exhibited genetic distances < 0.01. The remaining six species were *Chrysipterabiocellata* (up to 0.012 in genetic distances), *C.cyanea* (0.023), *C.rex* (0.023), *Neopomacentruscyanomos* (0.018), *Pomacentrustaeniometopon* (0.026) and *P.* sp. minamiiso (0.023). Amongst these species, *C.rex*, *P.taeniometopon* and *P.* sp minamiiso formed respective mixture clades, whereas the remaining three species formed respective unique clades in the NJ analysis.

The average number of base substitutions in the interspecific comparisons within same genera was 9.2–94.7 (0.016–0.126 in the p-distance), ranging from 0 to 138 bp (0–0.184 in the p-distance). Four genera, viz., *Amphiprion*, *Chromis*, *Plectroglyphidodon* and *Pomacentrus*, contained several pairs of species with a p-distance < 0.01.

Fig. 4 provides an overview of genetic distances for the MiFish region according to the third dataset that includes the values obtained from the second dataset. The average number of base substitutions amongst conspecific sequences was 0.8 bp (0.004 in the p-distance), ranging from 0 to 8 bp (0–0.048 in the p-distance). Of the 87 species for which sequences of more than one individual were available, 70 species (80.5%) exhibited sequence variations with genetic distances < 0.01. At the between-congeneric species level, the base substitutions were 0–41 bp, being equivalent to 0–0.248 of the uncorrected p-distance. These values were larger than those in the longer 12S dataset for most genera examined. Three genera, viz., *Amphiprion*, *Chrysiptera* and *Pomacentrus*, contained several pairs of species with p-distance < 0.01.

In the longer 12S dataset, 29 of 97 single-specimen species exhibited genetic distances > 0, but < 0.01 with the closest species, suggesting that their sequence differences from other species were not sufficiently large for stable and accurate identification. In the MiFish datasets with the same sample series, 16 of 97 single-specimen species exhibited genetic distances > 0, but < 0.01 from the respective closest species. In the third dataset, 20 of 96 single-specimen species exhibited genetic distances > 0, but < 0.01 from the respective closest species (Table 2). These species that fell below the threshold could be discriminated from any other species according to the sequences in the present datasets, but they were judged as species that require special cautions in identification, based on DNA barcoding.

## Discussion

Accurate species identification using DNA barcoding can be achieved when sufficient sequence differences exist between all pairwise combinations of species in the targeted DNA region (Ward 2009). Whether sequence differences exist between species depends on the region and length of the DNA fragments used and, therefore, we investigated sequence differences amongst pomacentrid species in two DNA regions, viz. the first half of mitochondrial 12S DNA (725–773 bp) and the MiFish region (166–199 bp). Our analyses confirmed that > 96.0% of the 150 pomacentrid species examined in this study can be discriminated by sequences of the longer 12S DNA region. Although our evaluation of sequence differences for single-specimen species using genetic distance cautioned that the degree of sequence difference of 31 species (20.7% of all species examined) is not sufficiently large for reliable species identification, it is useful for the tentative identification of these species. Exceptions are three pairs of species, viz. *Abudefdufsexfasciatus* and *A.vaigiensis*, *Pomacentruscaeruleus* and *P.caeruleopunctatus* and *P.taeniometopon* and *P.* sp. minamiiso, which share at least one identical sequence.

When the MiFish dataset was used for the same 150 species, an additional five pairs and two groups of species could not be discriminated from each other because of the lack of variable sites (Table 2) and 24 of the 150 species (16.0%) could not be identified. An additional 16 species exhibited genetic distances < 0.01 from the respective closest relatives in the MiFish region and their identification requires some caution. Therefore, the longer 12S region exhibits better performance in discriminating pomacentrid species. However, the MiFish region has a merit for a higher probability of successful PCR amplification, especially when the quality of template DNAs is low (Deagle et al. 2006, Bylemans et al. 2018, Wei et al. 2018). Our analysis on the third dataset suggested that we could identify 83.6% of pomacentrid species to the species level using the MiFish region. This hypervariable region is useful in eDNA metabarcoding for pomacentrid fishes with some caution, as shown in Table 2.

Another merit of the MiFish region is a greater number of sufficient sequence references. Currently, we could find 1.5 times more sequences of 1.2 times more species of Pomacentridae in INSD than that in the longer 12S region. This advantage would be significant in accurate species identification, compensating for its possible limitation of less variable sites.

A possible failure of species identification might also arise from genetic introgression through hybridisation (Ward et al. 2009, Collins and Cruickshank 2013). In that case, the barcoding does not function well for the involved species irrespective of the length and region of DNA. In pomacentrid fishes, hybridisation has been reported for *Abudefdufvaigienis* and *A.sexfasciatus* (Montanari et al. 2016, Bertrand et al. 2017). Bertrand et al. (2017) demonstrated that these two species could be discriminated using nuclear DNA markers, but shared identical sequences in the mitochondrial cytochrome b gene in a broad geographical area, suggesting extensive mtDNA introgression. The absence of sequence differences between these two species in our datasets appears to be this case. Nevertheless, hybridisation appears to be rare amongst benthic nesting fishes, including the members of Pomacentridae, that engage in deliberate pair spawning and parental care for the eggs laid on a substrate until hatching (Yaakub et al. 2006). Only a few cases of hybridisation have been reported for the pomacentrid fish (e.g. van Herwerden and Doherty (2006), Coleman et al. (2014), He et al. (2019)). Therefore, the adverse effects of hybridisation on DNA barcoding for this group are considered limited.

Another source of obstacles in DNA barcoding is taxonomic confusion. Our examinations for DNA sequence differences amongst pomacentrid fish revealed taxonomic confusion in *P.taeniometopon* and its relatives. Motomura (2020) recognised a possibly undescribed species of the genus *Pomacentrus* from the Japanese waters, which is referred to as *Pomacentrus* sp. minamiiso. According to Nakabou (2013), *P.* sp minamiiso morphologically resembles *P.taeniometopon*, but is distinguished from the latter in having pointed and denser suborbital protrusions. Kato (2011) mentioned that *P.* sp minamiiso differs from *P.taeniometopon* in having a black spot at the top of the operculum. We recognised four morphotypes in our specimens collected from Okinawan waters, viz. 1) typical *P.taeniometopon* that lacks both pointed and denser suborbital protrusions and black spot at the top of the operculum, 2) a form having both, 3) a form having pointed and denser suborbital protrusions only and 4) a form having a black spot at the top of the operculum only (Table 1). Our DNA analysis supported recognitions of the first and third forms as respective clades, but the remaining two were intermixed with each other. The NJ tree constructed, based on the MiFish region with larger samples, further suggested that another species, *P.adelus*, was included in this species complex (Fig. 3). Further taxonomic studies are warranted for this group.

Another case of possible taxonomic confusion involves *Chrysipterarex* and its relatives. Our analyses demonstrated that the genetic distance within *C.rex* was very large up to 0.023 in the longer 12S dataset and 0.048 in the MiFish dataset. Two morphologically resembling species, *C.chysocephala* and *C.caesifrons*, were described previously (Allen et al. 2015). According to the sequences obtained from INSD, *C.rex* and *C.chysocephala* are confused with each other (Fig. 3). Although two sequences (FJ616332 and JN935816), labelled as *C.rex*, appear to be *C.chysocephala* or *C.caesifrons*, we refrain from replacing their labels because of the lack of any information concerning voucher and locality for these sequences. It is desired to revise the label of the relevant sequences in INSD for future DNA barcoding.

The family Pomacentridae is a diversified group containing more than 430 extant species (McCord et al. 2021). Our examinations for sequence variations in the longer 12S and MiFish regions of the mitochondrial DNA confirmed that 96.0% and 83.6%, respectively, of the examined species can be identified to the species level, demonstrating the effectiveness and limitation of DNA barcoding for Pomacentridae using these DNA regions. Our study also revealed more sequence references for the MiFish region, suggesting the possible advantage of this region in DNA barcoding (Weigand et al. 2019). In contrast, the longer 12S region is advantageous in terms of higher resolution in species discrimination. Considering that the latter region contains the MiFish region inside, we recommend the accumulation of longer sequences unless the cost required for this is much different. DNA barcoding, using mitochondrial DNA, is potentially interfered with pseudogenes or numts, and one of the steps to avoid such a confusion is careful comparisons with closely-related published sequences (Song et al. 2008). Gold et al. (2021) indicated that it is meaningful to expand the DNA database for a particular regional fauna in a step-by-step manner. The sequences we provided in this study contribute to the expansion of references for the Japanese Pomacentridae. Further studies are required for filling the gap of taxon sampling for more successful DNA barcoding.

## Figures and Tables

**Table 1.** List of the sequences we obtained by sequencing and from International Nucleotide Sequence Database (INSD). Sequences with voucher numbers are those determined here, based on specimens examination. Sequences without any marks in the columns of Datasets 1–3 are those taken from INSD, but excluded after scrutinisation for certainty. Note that species names of some sequences taken from INSD were revised in this list following Tang et al. (2021) taxonomic revision and that the original names deposited in INSD are given in the rightmost column

**Genus**	**Species**	**Voucher number**	**GenBank accession codes**	**Dataset 1**	**Dataset 2**	**Dataset 3**	**Original names deposited in INSD**
*Abudefduf* Fabricius, 1775	*A.bengalensis* (Bloch, 1787)		FJ616289	〇	〇	〇	
	*A.bengalensis* (Bloch, 1787)		LC104614			〇	
	*A.bengalensis* (Bloch, 1787)		LC519419			〇	
	*A.bengalensis* (Bloch, 1787)		LC579947			〇	
	*A.bengalensis* (Bloch, 1787)		LC584265			〇	
	*A.bengalensis* (Bloch, 1787)		LC589596			〇	
	*A.bengalensis* (Bloch, 1787)		LC589597				
	*A.bengalensis* (Bloch, 1787)		LC669510	〇	〇	〇	
	*A.bengalensis* (Bloch, 1787)		MH678614	〇	〇	〇	
	*A.caudobimaculatus* Okada & Ikeda, 1939	OCF-P10652	LC814855	〇	〇	〇	
	*A.caudobimaculatus* Okada & Ikeda, 1939	OCF-P10653	LC814856	〇	〇	〇	
	*A.caudobimaculatus* Okada & Ikeda, 1939	POM-R033	LC814857	〇	〇	〇	
	*A.caudobimaculatus* Okada & Ikeda, 1939	POM-R038	LC814858	〇	〇	〇	
	*A.hoefleri* (Steindachner, 1881)		FJ616290	〇	〇	〇	
	*A.notatus* (Day, 1870)		FJ616292	〇	〇	〇	
	*A.notatus* (Day, 1870)		LC146252			〇	
	*A.notatus* (Day, 1870)		LC589567			〇	
	*A.notatus* (Day, 1870)		LC589581			〇	
	*A.notatus* (Day, 1870)		LC650575	〇	〇	〇	
	*A.notatus* (Day, 1870)		LC654182	〇	〇	〇	
	*A.notatus* (Day, 1870)	POM-R029	LC814859	〇	〇	〇	
	*A.saxatilis* (Linnaeus, 1758)		AF285920			〇	
	*A.saxatilis* (Linnaeus, 1758)		AY279570	〇	〇	〇	
	*A.saxatilis* (Linnaeus, 1758)		FJ616293	〇	〇	〇	
	*A.septemfasciatus* (Cuvier, 1830)		FJ616294	〇	〇	〇	
	*A.septemfasciatus* (Cuvier, 1830)		LC146253			〇	
	*A.septemfasciatus* (Cuvier, 1830)		LC589580			〇	
	*A.septemfasciatus* (Cuvier, 1830)		LC589608			〇	
	*A.septemfasciatus* (Cuvier, 1830)		MZ597977			〇	
	*A.septemfasciatus* (Cuvier, 1830)	OCF-P10596	LC814860	〇	〇	〇	
	*A.sexfasciatus* (Lacepède, 1801)		AF285921			〇	
	*A.sexfasciatus* (Lacepède, 1801)		FJ616295	〇	〇	〇	
	*A.sexfasciatus* (Lacepède, 1801)		JN935809	〇	〇	〇	
	*A.sexfasciatus* (Lacepède, 1801)		LC146260			〇	
	*A.sexfasciatus* (Lacepède, 1801)		LC557080			〇	
	*A.sexfasciatus* (Lacepède, 1801)		LC589574			〇	
	*A.sexfasciatus* (Lacepède, 1801)		MZ598010			〇	
	*A.sexfasciatus* (Lacepède, 1801)	OCF-P10592	LC814861	〇	〇	〇	
	*A.sexfasciatus* (Lacepède, 1801)	OCF-P10613	LC814862	〇	〇	〇	
	*A.sexfasciatus* (Lacepède, 1801)	OCF-P10656	LC814863	〇	〇	〇	
	*A.sexfasciatus* (Lacepède, 1801)	OCF-P10657	LC814864	〇	〇	〇	
	*A.sordidus* (Forsskål, 1775)		FJ616296	〇	〇	〇	
	*A.sordidus* (Forsskål, 1775)		LC021279			〇	
	*A.sordidus* (Forsskål, 1775)		LC327122			〇	
	*A.sordidus* (Forsskål, 1775)		LC458258			〇	
	*A.sordidus* (Forsskål, 1775)		LC519416			〇	
	*A.sordidus* (Forsskål, 1775)		LC519417			〇	
	*A.sordidus* (Forsskål, 1775)		LC557081			〇	
	*A.sordidus* (Forsskål, 1775)		LC589571			〇	
	*A.sordidus* (Forsskål, 1775)		LC589609			〇	
	*A.sordidus* (Forsskål, 1775)		LC604990	〇	〇	〇	
	*A.sordidus* (Forsskål, 1775)		MZ598431			〇	
	*A.sparoides* (Quoy & Gaimard, 1825)		JQ707028	〇	〇	〇	
	*A.taurus* (Müller & Troschel, 1848)		FJ616297	〇	〇	〇	
	*A.vaigiensis* (Quoy & Gaimard, 1825)		AB969974			〇	
	*A.vaigiensis* (Quoy & Gaimard, 1825)		AP006016	〇	〇	〇	
	*A.vaigiensis* (Quoy & Gaimard, 1825)		KF374999	〇	〇	〇	
	*A.vaigiensis* (Quoy & Gaimard, 1825)		LC069664			〇	
	*A.vaigiensis* (Quoy & Gaimard, 1825)		LC069665			〇	
	*A.vaigiensis* (Quoy & Gaimard, 1825)		LC421703			〇	
	*A.vaigiensis* (Quoy & Gaimard, 1825)		LC500710			〇	
	*A.vaigiensis* (Quoy & Gaimard, 1825)		LC506679			〇	
	*A.vaigiensis* (Quoy & Gaimard, 1825)		LC519418			〇	
	*A.vaigiensis* (Quoy & Gaimard, 1825)		LC589595			〇	
	*A.vaigiensis* (Quoy & Gaimard, 1825)		LC669501	〇	〇	〇	
	*A.vaigiensis* (Quoy & Gaimard, 1825)		MH248164				
	*A.vaigiensis* (Quoy & Gaimard, 1825)		MT954104	〇	〇	〇	
	*A.vaigiensis* (Quoy & Gaimard, 1825)	OCF-P10628	LC814865	〇	〇	〇	
	*A.vaigiensis* (Quoy & Gaimard, 1825)	OCF-P10629	LC814866	〇	〇	〇	
	*A.vaigiensis* (Quoy & Gaimard, 1825)	OCF-P10654	LC814867	〇	〇	〇	
	*A.vaigiensis* (Quoy & Gaimard, 1825)	OCF-P10655	LC814868	〇	〇	〇	
	*A.whitleyi* Allen & Robertson, 1974		FJ616298	〇	〇	〇	
*Acanthochromis* Gill, 1863	*A.polyacanthus* (Bleeker, 1855)		FJ616299	〇	〇	〇	
	*A.polyacanthus* (Bleeker, 1855)		LC278119			〇	
	*A.polyacanthus* (Bleeker, 1855)		MN998653				
*Altrichthys* Allen, 1999	*A.curatus* Allen, 1999		FJ616300				
*Amblyglyphidodon* Bleeker, 1877	*A.aureus* (Valenciennes 1830)		AF285922				
	*A.aureus* (Valenciennes 1830)		FJ616301	〇	〇	〇	
	*A.curacao* (Bloch, 1787)		FJ616302	〇	〇	〇	
	*A.curacao* (Bloch, 1787)		LC021278			〇	
	*A.curacao* (Bloch, 1787)		LC146257				
	*A.curacao* (Bloch, 1787)		MH995533	〇	〇	〇	
	*A.curacao* (Bloch, 1787)	OCF-P10614	LC814869	〇	〇	〇	
	*A.curacao* (Bloch, 1787)	POM-R021	LC814870	〇	〇	〇	
	*A.leucogaster* (Bleeker, 1847)		AY279568	〇	〇	〇	
	*A.leucogaster* (Bleeker, 1847)		FJ616303	〇	〇	〇	
	*A.leucogaster* (Bleeker, 1847)		LC091988			〇	
	*A.orbicularis* (Hombron & Jacquinot, 1853)		JQ707029	〇	〇	〇	
	*A.ternatensis* (Bleeker, 1853)		JQ729316			〇	
*Amblypomacentrus* Bleeker, 1877	*A.annulatus* (Peters, 1855)		JQ729317	〇	〇	〇	*Chrysipteraannulata*
	*A.clarus* Allen & Adrim, 2000		FJ616304	〇	〇	〇	
	*A.kuiteri* (Allen & Rajasuriya, 1995)		FJ616336	〇	〇	〇	*Chrysipterakuiteri*
	*A.tricinctus* (Allen & Randall, 1974)		JQ729318			〇	*Chrysipteratricincta*
*Amphiprion* Bloch & Schneider, 1801	*A.akallopisos* Bleeker, 1853		AB979273	〇	〇	〇	
	*A.akindynos* Allen, 1972		JN935810	〇	〇	〇	
	*A.biaculeatus* (Bloch, 1790)		LC089001	〇	〇	〇	*Premnasbiaculeatus*
	*A.biaculeatus* (Bloch, 1790)		LC089038	〇	〇	〇	*Premnasbiaculeatus*
	*A.bicinctus* Rüppell, 1830		JQ030887	〇	〇	〇	
	*A.chrysopterus* Cuvier, 1830		MZ598387			〇	
	*A.chrysopterus* Cuvier, 1830		MZ598389			〇	
	*A.clarkii* (Bennett, 1830)		AB969975			〇	
	*A.clarkii* (Bennett, 1830)		AB979449	〇	〇	〇	
	*A.clarkii* (Bennett, 1830)		AF285923			〇	
	*A.clarkii* (Bennett, 1830)		FJ616305				
	*A.clarkii* (Bennett, 1830)		KJ174498	〇	〇	〇	
	*A.clarkii* (Bennett, 1830)	OCF-P10601	LC814871	〇	〇	〇	
	*A.clarkii* (Bennett, 1830)	POM-R032	LC814872	〇	〇	〇	
	*A.ephippium* (Bloch, 1790)		AB979272	〇	〇	〇	
	*A.frenatus* Brevoort, 1856		FJ616306	〇	〇	〇	
	*A.frenatus* Brevoort, 1856		KJ833752	〇	〇	〇	
	*A.frenatus* Brevoort, 1856		LC089039	〇	〇	〇	
	*A.frenatus* Brevoort, 1856		LC146255			〇	
	*A.frenatus* Brevoort, 1856	POM-R172	LC814873	〇	〇	〇	
	*A.melanopus* Bleeker, 1852		FJ616307	〇	〇	〇	
	*A.ocellaris* Cuvier, 1830		AP006017	〇	〇	〇	
	*A.ocellaris* Cuvier, 1830		FJ616308	〇	〇	〇	
	*A.ocellaris* Cuvier, 1830		OK326863	〇	〇	〇	
	*A.ocellaris* Cuvier, 1830	OCF-P10595	LC814874	〇	〇	〇	
	*A.ocellaris* Cuvier, 1830	OCF-P10612	LC814875	〇	〇	〇	
	*A.percula* (Lacepède, 1802)		AB979450	〇	〇	〇	
	*A.percula* (Lacepède, 1802)		AF285924				
	*A.percula* (Lacepède, 1802)		KJ174497	〇	〇	〇	
	*A.perideraion* Bleeker, 1855		FJ616309	〇	〇	〇	
	*A.perideraion* Bleeker, 1855		KJ833753	〇	〇	〇	
	*A.perideraion* Bleeker, 1855	OCF-P10602	LC814876	〇	〇	〇	
	*A.polymnus* (Linnaeus, 1758)		AB979695	〇	〇	〇	
	*A.polymnus* (Linnaeus, 1758)		KJ101554	〇	〇	〇	
	*A.sandaracinos* Allen, 1972		FJ616310	〇	〇	〇	
	*A.sandaracinos* Allen, 1972	OCF-P10649	LC814877	〇	〇	〇	
	*A.sebae* Bleeker, 1853		AB979696				
*Azurina* Jordan & McGregor, 1898	*A.cyanea* (Poey, 1860)		AF285925			〇	*Chromiscyanea*
	*A.cyanea* (Poey, 1860)		JQ707033	〇	〇	〇	*Chromiscyanea*
	*A.elerae* (Fowler & Bean, 1928)		LC579141			〇	*Chromiselerae*
	*A.hirundo* Jordan & McGrego, 1898		AY098623			〇	
	*A.hirundo* Jordan & McGrego, 1898		FJ616311	〇	〇	〇	
	*A.lepidolepis* (Bleeker, 1876)		JQ707037	〇	〇	〇	*Chromislepidolepis*
	*A.lepidolepis* (Bleeker, 1876)		LC104638			〇	*Chromislepidolepis*
	*A.multilineata* (Guichenot, 1853)		FJ616319	〇	〇	〇	*Chromismultilineata*
*Cheiloprion* Weber, 1913	*C.labiatus* (Day, 1877)		FJ616312	〇	〇	〇	
	*C.labiatus* (Day, 1877)		LC104627			〇	
*Chromis* Cuvier, 1814	*C.albicauda* Allen & Erdmann, 2009		LC069651				
	*C.albicauda* Allen & Erdmann, 2009		LC499384			〇	
	*C.albomaculata* Kamohara, 1960		LC579140			〇	
	*C.alpha* Randall, 1988		FJ616313	〇	〇	〇	
	*C.alpha* Randall, 1988		MZ598457			〇	
	*C.alta* Greenfield & Woods, 1980		JQ707030	〇	〇	〇	
	*C.analis* (Cuvier, 1830)		FJ616315				
	*C.analis* (Cuvier, 1830)		LC069650				
	*C.atripectoralis* Welander & Schultz, 1951		FJ616316	〇	〇	〇	
	*C.atripectoralis* Welander & Schultz, 1951		MH049003				
	*C.atripectoralis* Welander & Schultz, 1951		MZ597940			〇	
	*C.chromis* (Linnaeus, 1758)		FJ616317	〇	〇	〇	
	*C.chrysura* (Bliss, 1883)		LC104613			〇	
	*C.chrysura* (Bliss, 1883)	POM-R022	LC814878	〇	〇	〇	
	*C.cinerascens* (Cuvier, 1830)		LC104616			〇	
	*C.enchrysurus* Jordan & Gilbert, 1882		JQ707036	〇	〇	〇	*Chromisenchrysura*
	*C.flavapicis* Randall, 2001		MZ598165			〇	
	*C.fumea* (Tanaka, 1917)		LC069660			〇	
	*C.fumea* (Tanaka, 1917)		LC327121			〇	
	*C.katoi* Iwatsubo & Motomura, 2018		LC499559			〇	
	*C.mirationis* Tanaka, 1917		AB974585			〇	
	*C.mirationis* Tanaka, 1917		LC069646			〇	
	*C.mirationis* Tanaka, 1917		LC649139	〇	〇	〇	
	*C.notata* (Temminck & Schlegel, 1843)		AB969956			〇	
	*C.notata* (Temminck & Schlegel, 1843)		LC104631			〇	
	*C.notata* (Temminck & Schlegel, 1843)		LC340187			〇	
	*C.notata* (Temminck & Schlegel, 1843)		LC474195			〇	
	*C.notata* (Temminck & Schlegel, 1843)		LC474196			〇	
	*C.notata* (Temminck & Schlegel, 1843)		LC496195			〇	
	*C.notata* (Temminck & Schlegel, 1843)		LC496196			〇	
	*C.notata* (Temminck & Schlegel, 1843)		LC519420			〇	
	*C.notata* (Temminck & Schlegel, 1843)		LC597204	〇	〇	〇	
	*C.okamurai* Yamakawa & Randall, 1989		LC499303			〇	
	*C.opercularis* (Günther, 1867)		FJ616320	〇	〇	〇	
	*C.punctipinnis* (Cooper, 1863)		FJ616322	〇	〇	〇	
	*C.randalli* Greenfield & Hensley, 1970		MK100717			〇	
	*C.ternatensis* (Bleeker, 1856)		FJ616324	〇	〇	〇	
	*C.viridis* (Cuvier, 1830)		FJ616325	〇	〇	〇	
	*C.viridis* (Cuvier, 1830)		JN935818				
	*C.viridis* (Cuvier, 1830)		MZ597951			〇	
	*C.viridis* (Cuvier, 1830)	OCF-P10606	LC814879	〇	〇	〇	
	*C.viridis* (Cuvier, 1830)	OCF-P10607	LC814880	〇	〇	〇	
	*C.viridis* (Cuvier, 1830)	OCF-P10608	LC814881	〇	〇	〇	
	*C.weberi* Fowler & Bean, 1928		FJ616326	〇	〇	〇	
	*C.weberi* Fowler & Bean, 1928		LC104618			〇	
	*C.xanthochira* (Bleeker, 1851)		FJ616327	〇	〇	〇	
	*C.xanthura* (Bleeker, 1854)		FJ616328	〇	〇	〇	
	*C.xanthura* (Bleeker, 1854)		LC104634			〇	
	*C.xanthura* (Bleeker, 1854)		MZ598168			〇	
	*C.yamakawai* Iwatsubo & Motomura, 2013		AB969957			〇	
	*C.yamakawai* Iwatsubo & Motomura, 2013		AB969958			〇	
	*C.yamakawai* Iwatsubo & Motomura, 2013		LC069659			〇	
	*C.yamakawai* Iwatsubo & Motomura, 2013		LC499560			〇	
	*C.yamakawai* Iwatsubo & Motomura, 2013		LC645289			〇	
*Chrysiptera* Swainson, 1839	*C.biocellata* (Quoy & Gaimard, 1825)		JQ707040				
	*C.biocellata* (Quoy & Gaimard, 1825)		KP676936	〇	〇	〇	
	*C.biocellata* (Quoy & Gaimard, 1825)		LC104611				
	*C.biocellata* (Quoy & Gaimard, 1825)		LC146256			〇	
	*C.biocellata* (Quoy & Gaimard, 1825)		LC146258			〇	
	*C.biocellata* (Quoy & Gaimard, 1825)	OCF-P10637	LC814882	〇	〇	〇	
	*C.biocellata* (Quoy & Gaimard, 1825)	OCF-P10638	LC814883	〇	〇	〇	
	*C.biocellata* (Quoy & Gaimard, 1825)	OCF-P10670	LC814884	〇	〇	〇	
	*C.biocellata* (Quoy & Gaimard, 1825)	POM-R018	LC814885	〇	〇	〇	
	*C.caeruleolineata* (Allen, 1973)		LC557082			〇	
	*C.caeruleolineata* (Allen, 1973)		LC579139			〇	
	*C.chrysocephala* Manica, Pilcher & Oakley, 2002		LC579142			〇	
	*C.cyanea* (Quoy & Gaimard, 1825)		FJ616330	〇	〇	〇	
	*C.cyanea* (Quoy & Gaimard, 1825)		LC021276			〇	
	*C.cyanea* (Quoy & Gaimard, 1825)	OCF-P10600	LC814886	〇	〇	〇	
	*C.cyanea* (Quoy & Gaimard, 1825)	POM-R025	LC814887	〇	〇	〇	
	*C.cyanea* (Quoy & Gaimard, 1825)	POM-R220	LC814888	〇	〇	〇	
	*C.cyanea* (Quoy & Gaimard, 1825)	POM-R221	LC814889	〇	〇	〇	
	*C.cyanea* (Quoy & Gaimard, 1825)	POM-R222	LC814890	〇	〇	〇	
	*C.galba* (Allen & Randall, 1974)		MK100718			〇	
	*C.glauca* (Cuvier, 1830)		JQ707041	〇	〇	〇	
	*C.glauca* (Cuvier, 1830)		LC069654			〇	
	*C.glauca* (Cuvier, 1830)		LC091981			〇	
	*C.glauca* (Cuvier, 1830)	OCF-P10599	LC814891	〇	〇	〇	
	*C.glauca* (Cuvier, 1830)	POM-R024	LC814892	〇	〇	〇	
	*C.glauca* (Cuvier, 1830)	POM-R049	LC814893	〇	〇	〇	
	*C.leucopoma* (Cuvier, 1830)		FJ616329	〇	〇	〇	*Chrysipterabrownriggii*
	*C.leucopoma* (Cuvier, 1830)		LC091982			〇	*Chrysipterabrownriggii*
	*C.leucopoma* (Cuvier, 1830)		LC091983			〇	
	*C.leucopoma* (Cuvier, 1830)	OCF-P10636	LC814894	〇	〇	〇	
	*C.leucopoma* (Cuvier, 1830)	POM-R036	LC814895	〇	〇	〇	
	*C.oxycephala* (Bleeker, 1876)		FJ616331	〇	〇	〇	
	*C.parasema* (Fowler, 1918)		LC104633			〇	
	*C.rapanui* (Greenfield & Hensley, 1970)		MK100719			〇	
	*C.rex* (Snyder, 1909)		FJ616332	〇	〇	〇	
	*C.rex* (Snyder, 1909)		JN935816	〇	〇	〇	
	*C.rex* (Snyder, 1909)		LC069653			〇	
	*C.rex* (Snyder, 1909)	POM-R109	LC814896	〇	〇	〇	
	*C.rex* (Snyder, 1909)	POM-R155	LC814897	〇	〇	〇	
	*C.rollandi* (Whitley, 1961)		AY098624			〇	
	*C.rollandi* (Whitley, 1961)		FJ616333	〇	〇	〇	
	*C.springeri* (Allen & Lubbock, 1976)		FJ616334	〇	〇	〇	
	*C.starcki* (Allen, 1973)		LC069652			〇	
	*C.starcki* (Allen, 1973)		LC091984			〇	
	*C.starcki* (Allen, 1973)	OCF-P10632	LC814898	〇	〇	〇	
	*C.talboti* (Allen, 1975)		FJ616335	〇	〇	〇	
	*C.taupou* (Jordan & Seale, 1906)		JQ707042	〇	〇	〇	
	*C.traceyi* (Woods & Schultz, 1960)		JQ707043	〇	〇	〇	
	*C.unimaculata* (Cuvier, 1830)		FJ616337				
	*C.unimaculata* (Cuvier, 1830)		LC146259			〇	
	*C.unimaculata* (Cuvier, 1830)	OCF-P10681	LC814899	〇	〇	〇	
	*C.unimaculata* (Cuvier, 1830)	POM-R204	LC814900	〇	〇	〇	
	*C.unimaculata* (Cuvier, 1830)	POM-R205	LC814901	〇	〇	〇	
*Dascyllus* Cuvier, 1829	*D.aruanus* (Linnaeus, 1758)		AF285927				
	*D.aruanus* (Linnaeus, 1758)		FJ616338	〇	〇	〇	
	*D.aruanus* (Linnaeus, 1758)		LC069655			〇	
	*D.aruanus* (Linnaeus, 1758)		MZ598231			〇	
	*D.aruanus* (Linnaeus, 1758)	OCF-P10591	LC814902	〇	〇	〇	
	*D.aruanus* (Linnaeus, 1758)	OCF-P10603	LC814903	〇	〇	〇	
	*D.aruanus* (Linnaeus, 1758)	OCF-P10627	LC814904	〇	〇	〇	
	*D.melanurus* Bleeker, 1854		FJ616339	〇	〇	〇	
	*D.reticulatus* (Richardson, 1846)		FJ616340	〇	〇	〇	
	*D.reticulatus* (Richardson, 1846)	OCF-P10604	LC814905	〇	〇	〇	
	*D.trimaculatus* (Rüppell, 1829)		AY279569	〇	〇	〇	
	*D.trimaculatus* (Rüppell, 1829)		FJ616341	〇	〇	〇	
	*D.trimaculatus* (Rüppell, 1829)		JN935817	〇	〇	〇	
	*D.trimaculatus* (Rüppell, 1829)		ON556619	〇	〇	〇	
	*D.trimaculatus* (Rüppell, 1829)	OCF-P10605	LC814906	〇	〇	〇	
*Dischistodus* Gill, 1863	*D.chrysopoecilus* (Schlegel & Müller, 1840)		AY279567	〇	〇	〇	
	*D.chrysopoecilus* (Schlegel & Müller, 1840)		FJ616342	〇	〇	〇	
	*D.fasciatus* (Cuvier, 1830)		LC340188			〇	
	*D.melanotus* (Bleeker, 1858)		FJ616343	〇	〇	〇	
	*D.perspicillatus* (Cuvier, 1830)		AY098625			〇	
	*D.perspicillatus* (Cuvier, 1830)		FJ616344	〇	〇	〇	
	*D.perspicillatus* (Cuvier, 1830)		LC340189			〇	
	*D.prosopotaenia* (Bleeker, 1852)		FJ616345	〇	〇	〇	
	*D.prosopotaenia* (Bleeker, 1852)		LC104632				
	*D.pseudochrysopoceilus* (Allen & Robertson, 1974)		FJ616346	〇	〇	〇	
*Hemiglyphidodon* Bleeker, 1877	*H.plagiometopon* (Bleeker, 1852)		FJ616347	〇	〇	〇	
	*H.plagiometopon* (Bleeker, 1852)		LC104626			〇	
	*H.plagiometopon* (Bleeker, 1852)		LC327207			〇	
*Hypsypops* Gill, 1861	*H.rubicundus* (Girard, 1854)		AF285928				
	*H.rubicundus* (Girard, 1854)		FJ616348	〇	〇	〇	
*Lepidozygus* Günther, 1862	*L.tapeinosoma* (Bleeker, 1856)		FJ616349	〇	〇	〇	
	*L.tapeinosoma* (Bleeker, 1856)		MZ598113			〇	
*Mecaenichthys* Whitley, 1929	*M.immaculatus* (Ogilby, 1885)		AF285929	〇	〇	〇	
	*M.immaculatus* (Ogilby, 1885)		FJ616350	〇	〇	〇	
*Microspathodon* Günther, 1862	*M.chrysurus* (Cuvier, 1830)		AF285930	〇	〇	〇	
	*M.chrysurus* (Cuvier, 1830)		FJ616351	〇	〇	〇	
	*M.dorsalis* (Gill, 1862)		FJ616352	〇	〇	〇	
*Neoglyphidodon* Allen, 1991	*N.carlsoni* (Allen, 1975)		JQ707044	〇	〇	〇	
	*N.melas* (Valenciennes 1830)		FJ616353	〇	〇	〇	
	*N.melas* (Valenciennes 1830)		LC104621			〇	
	*N.nigroris* (Cuvier, 1830)		FJ616354	〇	〇	〇	
	*N.nigroris* (Cuvier, 1830)		LC104620			〇	
	*N.nigroris* (Cuvier, 1830)		MH049057				
	*N.nigroris* (Cuvier, 1830)	POM-R034	LC814907	〇	〇	〇	
	*N.oxyodon* (Bleeker, 1858)		FJ616355	〇	〇	〇	
	*N.polyacnthus* (Ogilby, 1889)		JQ707045	〇	〇	〇	
	*N.thoractaeniatus* (Fowler & Bean, 1928)		FJ616356	〇	〇	〇	
*Neopomacentrus* Allen,1975	*N.azysron* (Bleeker, 1877)		FJ616357	〇	〇	〇	
	*N.bankieri* (Richardson, 1846)		JN935812	〇	〇	〇	
	*N.cyanomos* (Bleeker, 1856)		AY098626				
	*N.cyanomos* (Bleeker, 1856)		JQ707046				
	*N.cyanomos* (Bleeker, 1856)		LC104630			〇	
	*N.cyanomos* (Bleeker, 1856)	OCF-P10623	LC814908	〇	〇	〇	
	*N.cyanomos* (Bleeker, 1856)	OCF-P10624	LC814909	〇	〇	〇	
	*N.cyanomos* (Bleeker, 1856)	OCF-P10625	LC814910	〇	〇	〇	
	*N.metallicus* (Jordan & Seale, 1906)		JQ707047	〇	〇	〇	
	*N.taeniurus* (Bleeker, 1856)		FJ616358	〇	〇	〇	
	*N.taeniurus* (Bleeker, 1856)		LC021280			〇	
	*N.taeniurus* (Bleeker, 1856)		LC458260			〇	
	*N.taeniurus* (Bleeker, 1856)	OCF-P10647	LC814911	〇	〇	〇	
	*N.taeniurus* (Bleeker, 1856)	OCF-P10648	LC814912	〇	〇	〇	
*Nexilosus* Heller & Snodgrass, 1903	*N.latifrons* (Tschudi, 1846)		FJ616359				
*Parma* Günther, 1862	*P.microlepis* Günther, 1862		FJ616360	〇	〇	〇	
	*P.oligolepis* Whitley, 1929		AF285932				
	*P.oligolepis* Whitley, 1929		JN935815	〇	〇	〇	
	*P.oligolepis* Whitley, 1929		JQ707048				
*Plectroglyphidodon* Fowler & Ball, 1924	*P.altus* (Okada & Ikeda, 1937)		LC069643			〇	*Stegastesfasciolatus*
	*P.altus* (Okada & Ikeda, 1937)		LC091986			〇	*Stegastesfasciolatus*
	*P.altus* (Okada & Ikeda, 1937)		LC499326				
	*P.altus* (Okada & Ikeda, 1937)	OCF-P10635	LC814914	〇	〇	〇	
	*P.altus* (Okada & Ikeda, 1937)	POM-R027	LC814913	〇	〇	〇	
	*P.apicalis* (De Vis, 1885)		FJ616384	〇	〇	〇	*Stegastesapicalis*
	*P.aureus* (Fowler, 1927)		MZ598139			〇	*Stegastesaureus*
	*P.dickii* (Liénard, 1839)		FJ616361	〇	〇	〇	
	*P.dickii* (Liénard, 1839)		JN935813	〇	〇	〇	
	*P.dickii* (Liénard, 1839)		LC104624			〇	
	*P.fasciolatus* (Ogilby, 1889)		FJ616386	〇	〇	〇	*Stegastesfasciolatus*
	*P.fasciolatus* (Ogilby, 1889)		LC104623				*Stegastesfasciolatus*
	*P.fasciolatus* (Ogilby, 1889)		MZ598286			〇	*Stegastesfasciolatus*
	*P.gascoynei* (Whitley, 1964)		JN935814	〇	〇	〇	*Stegastesgascoynei*
	*P.imparipennis* (Vaillant & Sauvage, 1875)		LC104622			〇	
	*P.imparipennis* (Vaillant & Sauvage, 1875)		MZ598285			〇	
	*P.imparipennis* (Vaillant & Sauvage, 1875)	OCF-P10597	LC814915	〇	〇	〇	
	*P.insularis* (Allen & Emery, 1985)		LC069645			〇	*Stegastesinsularis*
	*P.johnstonianus* (Fowler & Ball, 1924)		LC104636			〇	
	*P.leucozona* (Bleeker, 1859)		FJ616363	〇	〇	〇	*Plectroglyphidodonleucozonus*
	*P.leucozona* (Bleeker, 1859)		LC104628			〇	*Plectroglyphidodonleucozonus*
	*P.leucozona* (Bleeker, 1859)		MZ598163			〇	*Plectroglyphidodonleucozonus*
	*P.leucozonus* (Bleeker, 1859)	POM-R037	LC814916	〇	〇	〇	*Plectroglyphidodonleucozonus*
	*P.obreptus* (Whitley, 1948)		FJ616390	〇	〇	〇	*Stegastesobreptus*
	*P.phoenixensis* (Schultz, 1943)		MZ598430			〇	
*Pomacentrus* Lacepède, 1802	*P.adelus* Allen, 1991		LC499307			〇	
	*P.albicaudatus* Baschieri-Salvadori, 1955		FJ616364				
	*P.alexanderae* Evermann & Seale, 1907		FJ616365				
	*P.alexanderae* Evermann & Seale, 1907		LC104612			〇	
	*P.alexanderae* Evermann & Seale, 1907	OCF-P10615	LC814917	〇	〇	〇	
	*P.alexanderae* Evermann & Seale, 1907	OCF-P10630	LC814918	〇	〇	〇	
	*P.alexanderae* Evermann & Seale, 1907	POM-R020	LC814919	〇	〇	〇	
	*P.alleni* Burgess, 1981		JQ707049	〇	〇	〇	
	*P.amboinensis* Bleeker, 1868		FJ616366	〇	〇	〇	
	*P.amboinensis* Bleeker, 1868		JN935811	〇	〇	〇	
	*P.amboinensis* Bleeker, 1868		LC021277			〇	
	*P.amboinensis* Bleeker, 1868		LC278024			〇	
	*P.amboinensis* Bleeker, 1868	OCF-P10593	LC814920	〇	〇	〇	
	*P.amboinensis* Bleeker, 1868	OCF-P10594	LC814921	〇	〇	〇	
	*P.amboinensis* Bleeker, 1868	OCF-P10643	LC814922	〇	〇	〇	
	*P.auriventris* Allen, 1991		JQ707050	〇	〇	〇	
	*P.baenschi* Allen, 1991		JQ707051	〇	〇	〇	
	*P.bankanensis* Bleeker, 1854		FJ616367				
	*P.bankanensis* Bleeker, 1854		LC069663			〇	
	*P.bankanensis* Bleeker, 1854		LC104629				
	*P.bankanensis* Bleeker, 1854	OCF-P10639	LC814923	〇	〇	〇	
	*P.bankanensis* Bleeker, 1854	OCF-P10640	LC814924	〇	〇	〇	
	*P.bankanensis* Bleeker, 1854	OCF-P10669	LC814925	〇	〇	〇	
	*P.bankanensis* Bleeker, 1854	OCF-P10675	LC814926	〇	〇	〇	
	*P.bankanensis* Bleeker, 1854	POM-R051	LC814927	〇	〇	〇	
	*P.bankanensis* Bleeker, 1854	POM-R208	LC814928	〇	〇	〇	
	*P.bankanensis* Bleeker, 1854	POM-R209	LC814929	〇	〇	〇	
	*P.bankanensis* Bleeker, 1854	POM-R210	LC814930	〇	〇	〇	
	*P.bankanensis* Bleeker, 1854	POM-R211	LC814931	〇	〇	〇	
	*P.brachialis* Cuvier, 1830		FJ616368	〇	〇	〇	
	*P.burroughi* Fowler, 1918		FJ616369	〇	〇	〇	
	*P.caeruleopunctatus* Allen, 2002		JQ707052	〇	〇	〇	
	*P.caeruleus* Quoy & Gaimard, 1825		JQ707053	〇	〇	〇	
	*P.callainus* Randall, 2002		JQ707054	〇	〇	〇	
	*P.chrysurus* Cuvier, 1830		FJ616370	〇	〇	〇	
	*P.chrysurus* Cuvier, 1830		LC091985			〇	
	*P.chrysurus* Cuvier, 1830	OCF-P10641	LC814932	〇	〇	〇	
	*P.chrysurus* Cuvier, 1830	OCF-P10642	LC814933	〇	〇	〇	
	*P.chrysurus* Cuvier, 1830	OCF-P10667	LC814934	〇	〇	〇	
	*P.chrysurus* Cuvier, 1830	OCF-P10668	LC814935	〇	〇	〇	
	*P.chrysurus* Cuvier, 1830	POM-R026	LC814936	〇	〇	〇	
	*P.coelestis* Jordan & Starks, 1901		FJ616371	〇	〇	〇	
	*P.coelestis* Jordan & Starks, 1901		LC069662			〇	
	*P.coelestis* Jordan & Starks, 1901		LC579949			〇	
	*P.coelestis* Jordan & Starks, 1901		LC649221	〇	〇	〇	
	*P.coelestis* Jordan & Starks, 1901		MZ598164			〇	
	*P.coelestis* Jordan & Starks, 1901	OCF-P10609	LC814937	〇	〇	〇	
	*P.coelestis* Jordan & Starks, 1901	OCF-P10610	LC814938	〇	〇	〇	
	*P.coelestis* Jordan & Starks, 1901	OCF-P10634	LC814939	〇	〇	〇	
	*P.grammorhynchus* Fowler, 1918		FJ616372	〇	〇	〇	
	*P.imitator* (Whitley, 1964)		JQ707055	〇	〇	〇	
	*P.lepidogenys* Fowler & Bean, 1928		FJ616373	〇	〇	〇	
	*P.lepidogenys* Fowler & Bean, 1928		LC104609			〇	
	*P.lepidogenys* Fowler & Bean, 1928	OCF-P10678	LC814940	〇	〇	〇	
	*P.moluccensis* Bleeker, 1853		FJ616374				
	*P.moluccensis* Bleeker, 1853		LC091989			〇	
	*P.moluccensis* Bleeker, 1853		MT199209	〇	〇	〇	
	*P.moluccensis* Bleeker, 1853		MT410992	〇	〇	〇	
	*P.moluccensis* Bleeker, 1853	OCF-P10626	LC814941	〇	〇	〇	
	*P.moluccensis* Bleeker, 1853	OCF-P10651	LC814942	〇	〇	〇	
	*P.moluccensis* Bleeker, 1853	OCF-P10679	LC814943	〇	〇	〇	
	*P.moluccensis* Bleeker, 1853	OCF-P10680	LC814944	〇	〇	〇	
	*P.moluccensis* Bleeker, 1853	POM-R040	LC814945	〇	〇	〇	
	*P.nagasakiensis* Tanaka, 1917		LC069661			〇	
	*P.nigromanus* Weber, 1913		FJ616375				
	*P.nigromarginatus* Allen, 1973		FJ616376	〇	〇	〇	
	*P.pavo* (Bloch, 1787)		JQ707056				
	*P.pavo* (Bloch, 1787)		MZ598233			〇	
	*P.philippinus* Evermann & Seale, 1907		FJ616377				
	*P.philippinus* Evermann & Seale, 1907		LC146254			〇	
	*P.philippinus* Evermann & Seale, 1907	OCF-P10616	LC814946	〇	〇	〇	
	*P.philippinus* Evermann & Seale, 1907	OCF-P10617	LC814947	〇	〇	〇	
	*P.philippinus* Evermann & Seale, 1907	OCF-P10631	LC814948	〇	〇	〇	
	*P.* sp. 'minamiiso'		LC579138			〇	*Pomacentrus* sp. minami-iso-suzumedai
	*P.* sp. 'minamiiso'		LC579216			〇	*Pomacentrus* sp. minami-iso-suzumedai
	*P.* sp. 'minamiiso'	OCF-P10611	LC814955	〇	〇	〇	
	*P.* sp. 'minamiiso'	OCF-P10645	LC814956	〇	〇	〇	
	*P.* sp. 'minamiiso'	OCF-P10646	LC814957	〇	〇	〇	
	*P.* sp. 'minamiiso'	OCF-P10658	LC814958	〇	〇	〇	
	*P.* sp. 'minamiiso'	OCF-P10659	LC814959	〇	〇	〇	
	*P.* sp. 'minamiiso'	OCF-P10660	LC814960	〇	〇	〇	
	*P.* sp. 'minamiiso'	OCF-P10663	LC814961	〇	〇	〇	
	*P.* sp. 'minamiiso'	POM-R041	LC814962	〇	〇	〇	
	*P.* sp. 'minamiiso'	POM-R093	LC814963	〇	〇	〇	
	*P.* sp. 'minamiiso'	POM-R212	LC814964	〇	〇	〇	
	*P.* sp. 'minamiiso'	POM-R213	LC814965	〇	〇	〇	
	*P.* sp. 'minamiiso'	POM-R215	LC814966	〇	〇	〇	
	*P.* sp. 'minamiiso' (morphotype 'less spot')	OCF-P10618	LC814949	〇	〇	〇	
	*P.* sp. 'minamiiso' (morphotype 'less spot')	OCF-P10619	LC814950	〇	〇	〇	
	*P.* sp. 'minamiiso' (morphotype 'less spot')	OCF-P10620	LC814951	〇	〇	〇	
	*P.* sp. 'minamiiso' (morphotype 'less spot')	OCF-P10621	LC814952	〇	〇	〇	
	*P.* sp. 'minamiiso' (morphotype 'less spot')	OCF-P10622	LC814953	〇	〇	〇	
	*P.* sp. 'minamiiso' (morphotype 'less spot')	POM-R092	LC814954	〇	〇	〇	
	*P.stigma* Fowler & Bean, 1928		FJ616378	〇	〇	〇	
	*P.sulfreus* Klunzinger, 1871		JQ707057	〇	〇	〇	
	*P.taeniometopon* Bleeker, 1852		AB972176			〇	
	*P.taeniometopon* Bleeker, 1852		LC069648				
	*P.taeniometopon* Bleeker, 1852		LC091991			〇	
	*P.taeniometopon* Bleeker, 1852		LC458259			〇	
	*P.taeniometopon* Bleeker, 1852		LC552553			〇	
	*P.taeniometopon* Bleeker, 1852	OCF-P10650	LC814967	〇	〇	〇	
	*P.taeniometopon* Bleeker, 1852	POM-R023	LC814968	〇	〇	〇	
	*P.taeniometopon* Bleeker, 1852 (morphotype 'with spot')	OCF-P10661	LC814969	〇	〇	〇	
	*P.taeniometopon* Bleeker, 1852 (morphotype 'with spot')	OCF-P10662	LC814970	〇	〇	〇	
	*P.taeniometopon* Bleeker, 1852 (morphotype 'with spot')	OCF-P10664	LC814971	〇	〇	〇	
	*P.taeniometopon* Bleeker, 1852 (morphotype 'with spot')	OCF-P10665	LC814972	〇	〇	〇	
	*P.taeniometopon* Bleeker, 1852 (morphotype 'with spot')	OCF-P10666	LC814973	〇	〇	〇	
	*P.taeniometopon* Bleeker, 1852 (morphotype 'with spot')	OCF-P10672	LC814974	〇	〇	〇	
	*P.taeniometopon* Bleeker, 1852 (morphotype 'with spot')	OCF-P10673	LC814975	〇	〇	〇	
	*P.taeniometopon* Bleeker, 1852 (morphotype 'with spot')	OCF-P10674	LC814976	〇	〇	〇	
	*P.trilineatus* Cuvier, 1830		JQ707058	〇	〇	〇	
	*P.tripunctatus* Cuvier, 1830		LC104610			〇	
	*P.vaiuli* Jordan & Seale, 1906		AF285935			〇	
	*P.vaiuli* Jordan & Seale, 1906		FJ616379				
	*P.vaiuli* Jordan & Seale, 1906		LC069647			〇	
	*P.vaiuli* Jordan & Seale, 1906		LC091987			〇	
	*P.vaiuli* Jordan & Seale, 1906	OCF-P10633	LC814977	〇	〇	〇	
*Pomachromis* Allen & Randall, 1974	*P.fuscidorsalis* Allen & Randall, 1974		MZ597904			〇	
	*P.fuscidorsalis* Allen & Randall, 1974		MZ597957			〇	
	*P.richardsoni* (Snyder, 1909)		FJ616380	〇	〇	〇	
	*P.richardsoni* (Snyder, 1909)		LC091990			〇	
	*P.richardsoni* (Snyder, 1909)	OCF-P10671	LC814978	〇	〇	〇	
	*P.richardsoni* (Snyder, 1909)	POM-R039	LC814979	〇	〇	〇	
*Pristotis* Rüppell, 1838	*P.obtusirostris* (Günther, 1862)		FJ616382	〇	〇	〇	
	*P.obtusirostris* (Günther, 1862)		LC069641			〇	
	*P.obtusirostris* (Günther, 1862)		LC069649			〇	
*Pycnochromis* Fowler, 1941	*P.acares* (Randall & Swerdloff, 1973)		LC499306			〇	*Chromisacares*
	*P.acares* (Randall & Swerdloff, 1973)		MZ598241			〇	*Chromisacares*
	*P.agilis* (Smith, 1960)		LC499305			〇	*Chromisagilis*
	*P.agilis* (Smith, 1960)		MZ598390			〇	*Chromisagilis*
	*P.alleni* (Randall, Ida & Moyer, 1981)		LC104635			〇	*Chromisalleni*
	*P.amboinensis* (Bleeker, 1871)		FJ616314	〇	〇	〇	*Chromisamboinensis*
	*P.atripes* (Fowler & Bean, 1928)		JQ707031	〇	〇	〇	*Chromisatripes*
	*P.atripes* (Fowler & Bean, 1928)		LC069656			〇	*Chromisatripes*
	*P.atripes* (Fowler & Bean, 1928)		LC104637			〇	*Chromisatripes*
	*P.bami* (Randall & McCosker, 1992)		JQ707032				*Chromisbami*
	*P.delta* (Randall, 1988)		JQ707034	〇	〇	〇	*Chromisdelta*
	*P.dimidata* (Klunzinger, 1871)		JQ707035				*Chromisdimidata*
	*P.iomelas* (Jordan & Seale, 1906)		AF285926				*Chromisiomelas*
	*P.iomelas* (Jordan & Seale, 1906)		MZ598215				*Chromisiomelas*
	*P.leucurus* (Gilbert, 1905)		MZ598456			〇	*Chromisleucurus*
	*P.margaritifer* (Fowler, 1946)		FJ616318	〇	〇	〇	*Chromismargaritifer*
	*P.margaritifer* (Fowler, 1946)		LC104619			〇	*Chromismargaritifer*
	*P.margaritifer* (Fowler, 1946)		LC720453	〇	〇	〇	*Chromismargaritifer*
	*P.margaritifer* (Fowler, 1946)		MZ597994			〇	*Chromismargaritifer*
	*P.nigrurus* (Smith, 1960)		JQ707038	〇	〇	〇	*Chromisnigrura*
	*P.ovatiformis* (Fowler, 1946)		FJ616321	〇	〇	〇	*Chromisovatiformis*
	*P.ovatiformis* (Fowler, 1946)		LC091979				
	*P.ovatiformis* (Fowler, 1946)		LC091980			〇	
	*P.retrofasciatus* (Weber, 1913)		FJ616323	〇	〇	〇	*Chromisretrofasciata*
	*P.retrofasciatus* (Weber, 1913)		LC552562			〇	*Chromisretrofasciata*
	*P.retrofasciatus* (Weber, 1913)		MH049138				*Chromisretrofasciata*
	*P.vanderbilti* Fowler, 1941		JQ707039	〇	〇	〇	*Chromisvanderbilti*
	*P.vanderbilti* Fowler, 1941		LC104617			〇	*Chromisvanderbilti*
	*P.vanderbilti* Fowler, 1941		MZ598283			〇	*Chromisvanderbilti*
*Similiparma* Hensley, 1986	*S.hermani* (Steindachner, 1887)		FJ616383	〇	〇	〇	
	*S.lurida* (Cuvier, 1830)		FJ616291	〇	〇	〇	*Abudefdufluridus*
*Stegastes* Jenyns, 1840	*S.adustus* (Troschel, 1865)		AF285937				
	*S.adustus* (Troschel, 1865)		JQ707059	〇	〇	〇	
	*S.albifasciatus* (Schlegel & Müller, 1840)		JQ707060	〇	〇	〇	
	*S.albifasciatus* (Schlegel & Müller, 1840)		LC069644			〇	
	*S.albifasciatus* (Schlegel & Müller, 1840)		MZ597945			〇	
	*S.albifasciatus* (Schlegel & Müller, 1840)	OCF-P10676	LC814980	〇	〇	〇	
	*S.albifasciatus* (Schlegel & Müller, 1840)	POM-R030	LC814981	〇	〇	〇	
	*S.diencaeus* (Jordan & Rutter, 1897)		FJ616385				
	*S.flavilatus* (Gill, 1862)		JQ729319	〇	〇	〇	
	*S.flavilatus* (Gill, 1862)		KP136922	〇	〇	〇	
	*S.fuscus* (Cuvier, 1830)		JQ707061				
	*S.imbricatus* Jenyns, 1840		FJ616387	〇	〇	〇	
	*S.lacrymatus* (Quoy & Gaimard, 1825)		AF285933			〇	*Plectroglyphidodonlacrymatus*
	*S.lacrymatus* (Quoy & Gaimard, 1825)		FJ616362	〇	〇	〇	*Plectroglyphidodonlacrymatus*
	*S.lacrymatus* (Quoy & Gaimard, 1825)		LC104625			〇	*Plectroglyphidodonlacrymatus*
	*S.lacrymatus* (Quoy & Gaimard, 1825)	OCF-P10598	LC814982	〇	〇	〇	
	*S.leucosticus* (Müller & Troschel, 1848)		FJ616388	〇	〇	〇	
	*S.lividus* (Forster, 1801)		MZ597913			〇	
	*S.nigricans* (Lacepède, 1802)		FJ616389	〇	〇	〇	
	*S.nigricans* (Lacepède, 1802)		LC104615			〇	
	*S.nigricans* (Lacepède, 1802)		MZ597950			〇	
	*S.nigricans* (Lacepède, 1802)	OCF-P10644	LC814984	〇	〇	〇	
	*S.nigricans* (Lacepède, 1802)	OCF-P10677	LC814983	〇	〇	〇	
	*S.partius* (Poey, 1868)		FJ616391	〇	〇	〇	
	*S.planifrons* (Cuvier, 1830)		JQ707062	〇	〇	〇	
	*S.rectifraenus* (Gill, 1862)		JQ729320	〇	〇	〇	
*Teixeirichthys* Smith, 1953	*T.jordani* (Rutter, 1897)		FJ616392	〇	〇	〇	
	*T.jordani* (Rutter, 1897)		LC069642			〇	

**Table 2.** List of the species or species pairs classified as “Split”, “Merge”, “Mixture” and/or those with genetic distances < 0.01 from the closest relatives (indicated as “Cautions”). Species denoted by asterisk are those whose sequences were only included in the MiFish dataset.

**Genus**	**Longer 12S dataset**	**Additional MiFish dataset**
*Abudefduf* Fabricius, 1775	*A.sexfasciatus* (Lacepède, 1801) (Mixture) and *A.vaigiensis* (Quoy & Gaimard, 1825) (Mixture)	*A.sexfasciatus* (Lacepède, 1801) (Mixture) and *A.vaigiensis* (Quoy & Gaimard, 1825) (Mixture)
	*A.hoefleri* (Steindachner, 1881)(Cautions)	
*Amblyglyphidodon* Bleeker, 1877		*A.cracao* (Bloch, 1787) (Merge) and *A.orbicularis* (Hombron & Jacquinot, 1853) (Merge)
*Amblypomacentrus* Bleeker, 1877		*A.kuiteri* (Allen & Rajasuriya, 1995) (Cautions)
		*A.tricinctus* (Allen & Randall, 1974)* (Cautions)
*Amphiprion* Bloch & Schneider, 1801	*A.bicinctus* Rüppell, 1830 (Cautions)	*A.bicinctus* Rüppell, 1830 (Merge), *A.chrysopterus* Cuvier, 1830* (Merge), *A.perideraion* Bleeker, 1855 (Merge) and *A.sandaracinos* Allen, 1972 (Merge)
	*A.akallopisos* Bleeker, 1853 (Cautions)	*A.akallopisos* Bleeker, 1853 (Cautions)
	*A.akindynos* Allen, 1972 (Cautions)	*A.akindynos* Allen, 1972 (Cautions)
	*A.ephippium* (Bloch, 1790) (Cautions)	*A.ephippium* (Bloch, 1790) (Cautions)
	*A.melanopus* Bleeker, 1852 (Cautions)	*A.melanopus* Bleeker, 1852 (Cautions)
*Chromis* Cuvier, 1814	*C.xanthura* (Bleeker, 1854) (Cautions)	*C.xanthura* (Bleeker, 1854) (Mixture) with *C.opercularis* (Günther, 1867) (Merge)
	*C.opereularis* (Günther, 1867) (Cautions)	
	*C.weberi* Fowler & Bean, 1928 (Cautions)	
	*C.xanthochira* (Bleeker, 1851) (Cautions)	*C.xanthochira* (Bleeker, 1851) (Cautions)
		*C.katoi* Iwatsubo & Motomura, 2018* (Cautions)
*Chrysiptera* Swainson, 1839		*C.rex* (Snyder, 1909) (Mixture) with *C.chrysocephala* Manica, Pilcher & Oakley, 2002* (Merge)
	*C.talboti* (Allen, 1975) (Cautions)	*C.talboti* (Allen, 1975) (Cautions)
	*C.traceyi* (Woods & Schultz, 1960) (Cautions)	*C.traceyi* (Woods & Schultz, 1960) (Cautions)
		*C.rapanui* (Greenfield & Hensley, 1970)* (Cautions)
*Dischistodus* Gill, 1863		*D.prosopotaenia* (Bleeker, 1852) (Cautions)
*Neoglyphidodon* Allen, 1991	*N.thoractaeniatus* (Fowler & Bean, 1928) (Cautions)	
*Neopomacentrus* Allen,1975	*N.azysron* (Bleeker, 1877) (Cautions)	
	*N.metallicus* (Jordan & Seale, 1906) (Cautions)	
*Plectroglyphidodon* Fowler & Ball, 1924 and *Stegastes* Jenyns, 1840	*P.apicalis* (De Vis, 1885) (Cautions)	*P.apicalis* (De Vis, 1885) (Merge) and *P.obreptus* (Whitley, 1948) with *S.albifasciatus* (Schlegel & Müller, 1840) (Mixture)
	*P.fasciolatus* (Ogilby, 1889) (Cautions)	
	*P.gascoynei* (Whitley, 1964) (Cautions)	*P.gascoynei* (Whitley, 1964) (Cautions)
	*P.obreptus* (Whitley, 1948) (Cautions)	
*Pomacentrus* Lacepède, 1802	*P.caeruleopunctatus* Allen, 2002 (Merge) and *P.caeruleus* Quoy & Gaimard, 1825 (Merge)	*P.alleni* Burgess, 1981 (Merge), *P.caeruleopunctatus* Allen, 2002 (Merge) and *P.caeruleus* Quoy & Gaimard, 1825 (Merge) and *P.coelestis* Jordan & Starks, 1901 (Mixture)
	*P.alleni* Burgess, 1981 (Cautions)	
	*P.taeniometopon* Bleeker, 1852 (Mixture) and *P.* sp. 'minamiiso' (Mixture)	*P.adelus* Allen, 1991* (Merge), *P.taeniometopon* Bleeker, 1852 (Mixture) and *P.* sp. 'minamiiso' (Mixture)
	*P.stigma* Fowler & Bean, 1928 (Cautions)	*P.alexanderae* Evermann & Seale, 1907 (Merge) and *P.stigma* Fowler & Bean, 1928 (Merge)
		*P.philipinus* Evermann & Seale, 1907 (sprit)
		*P.vaiuli* Jordan & Seale, 1906 (sprit)
	*P.auriventris* Allen, 1991 (Cautions)	*P.auriventris* Allen, 1991 (Cautions)
	*P.brachialis* Cuvier, 1830 (Cautions)	
	*P.burroughi* Fowler, 1918 (Cautions)	*P.burroughi* Fowler, 1918 (Cautions)
	*P.callainus* Randall, 2002 (Cautions)	*P.callainus* Randall, 2002 (Cautions)
	*P.grammorhynchus* Fowler, 1918 (Cautions)	*P.grammorhynchus* Fowler, 1918 (Cautions)
	*P.imitator* (Whitley, 1964) (Cautions)	*P.imitator* (Whitley, 1964) (Cautions)
	*P.nigromarginatus* Allen, 1973 (Cautions)	
		*P.nagasakiensis* Tanaka, 1917* (Cautions)
		*P.sulfreus* Klunzinger, 1871 (Cautions)
*Pycnochromis* Fowler, 1941		*P.acares* (Randall & Swerdloff, 1973)* (Merge) and *P.nigrurus* (Smith, 1960) (Merge)
	*P.amboinensis* (Bleeker, 1871) (Cautions)	*P.amboinensis* (Bleeker, 1871) (Merge) and *P.ovatiformis* (Fowler, 1946) (Merge)
	*P.ovatiformis* (Fowler, 1946) (Cautions)	
